# Premature ejaculation: An investigative study into assumptions, facts and perceptions of patients from the Middle East (PEAP STUDY)

**DOI:** 10.1080/2090598X.2021.1948159

**Published:** 2021-07-09

**Authors:** Ahmad Albakr, Mohamed Arafa, Haitham Elbardisi, Sami ElSaid, Ahmad Majzoub

**Affiliations:** a Urology Department, Hamad Medical Corporation, Doha, Qatar; b Andrology Department, Cairo University, Cairo, Egypt; c Urology Department, Weill Cornell Medicine-Qatar, Doha, Qatar; d American Center for Reproductive Medicine, Cleveland, OH, USA

**Keywords:** Premature ejaculation, Qatar, perception, AIPE

## Abstract

**Objective:** To determine the prevalence of premature ejaculation (PE) in Qatar as a representative of the Middle East region and the population perception of normal ejaculation.

**Subjects and methods:** This study was a cross-sectional, observational, non-interventional, epidemiological study, conducted from February 2012 to February 2013. Randomly selected married males were asked to answer two questionnaires in a direct interview. The first questionnaire assessed the PE complaint, the time between ejaculation and intromission (actual intravaginal ejaculatory latency time [IELT-a]), and the perceived normal average time between intromission and ejaculation (IELT-p). The second questionnaire used was the Arabic Index of Premature Ejaculation (AIPE).

**Results:** A total of 3042 subjects were included. The mean (SD) age was 37.09 (9.1) years. The prevalence of PE in Qatar using the self-report and AIPE score was 38.5% and 36.2%, respectively. The median (interquartile range) IELT-a and IELT-p were 5 (3–13.5) and 15 (5–15) min. In the AIPE-confirmed PE group, and according to the AIPE severity classification, the differences in IELT-a and IELT-p between the severity groups were highly significant, with the duration of both IELT-a and ILET-p being higher in AIPE-No-PE and mild-PE groups (*P* < 0.001 for both). A negative correlation was found between AIPE score and age in the PE group.

**Conclusion:** The prevalence of PE in Qatar is high. PE prevalence was found to increase with age. The IELT and perception of normal IELT were both correlated with the severity of PE.

## Introduction

The evaluation and management of premature ejaculation (PE) have been subject to major obscurities in the past. Difficulty in reaching an accurate definition for PE was the principal reason behind this confusion. A global estimation of the weight of the problem remained unclear for a long time for the same reason. With the development of internationally recognised definitions through the American Psychiatric Association’s Diagnostic and Statistical Manual of Mental Disorders (DSM) III in 1980 and WHO International Classification of Diseases Tenth Edition (ICD-10) in 1994, a better understanding of the disease characteristics has been achieved. Nonetheless, the DSM-III could not objectively verify the disease and ICD-10 could not provide clear evidence of the abnormal ejaculation latency time [1]. The need for an evidence based standardised definition was met with the International Society for Sexual Medicine (ISSM) definition in 2013 and the DSM-V released in the same year [2].

Multiple patient-reported outcome tools (PROs) have been proposed to aid the diagnosis of PE. These tools are meant to assess the patient’s subjective perception of the disorder. Five tools have been validated to date, the Premature Ejaculation Profile (PEP), Index of Premature Ejaculation (IPE), Premature Ejaculation Diagnostic Tool (PEDT), The Chinese Index of Premature Ejaculation (CIPE) and the Arabic Index of Premature Ejaculation (AIPE) [3]. The AIPE has been used and validated in the Middle East with reliable outcomes. It consists of seven questions assessing sexual desire, erectile dysfunction, intravaginal ejaculatory latency time (IELT), control of ejaculation, sexual satisfaction, partner satisfaction, and associated psychogenic effects related to the sexual act [4].

In the shadow of the lack of standardised definitions, a wide range of conflicting results of PE prevalence have been reported [1]. Various methods have been used for assessing the disease epidemiology including patient interview, mailed questionnaires, and web-based surveys [5]. Early ejaculation was reported by the Global Study of Sexual Attitudes and Behaviors (GSSAB) as the most common sexual dysfunction in men from various ethnic and cultural backgrounds [6]. The study investigated 27500 subjects over 29 countries. It showed a prevalence of 8% (7–10%) of early ejaculation in the Middle East. In addition, it showed variable prevalence of early ejaculation between various ethnic and cultural backgrounds. The GSSAB had multiple limitations. First, the youngest included study subjects were aged 40 years, while PE can be seen before this age. The study population came from variable cultural backgrounds and accurate translation of the surveys was a challenge. Recruitment and sample differences between different countries were difficult and variable, with questionable adequacy of samples between countries. Finally, quality variation between the different survey organisations is a factor to consider [7].

To our knowledge no wide scale epidemiological studies have addressed the prevalence of PE in the Middle East other than the GSSAB. In addition, a search could not yield any papers discussing Middle Eastern patients’ perception of normal ejaculation. The PE investigative study on the assumptions, facts and perceptions of patients from the Middle East (PEAP STUDY) aimed to: (1) document the prevalence of PE in Qatar, (2) explore the characteristics of PE in Qatar, and (3) assess the perception of normal ejaculation time among men in Qatar. It is important to note that Qatar is a Gulf cosmopolitan country with a special population distribution. More than 75% of the Qatar population is composed of expatriates mainly from the Gulf area, Middle East and North Africa (MENA) region and South Asia. The remaining are Qatari nationals representing the Gulf community. For this reason, the Qatar population is a perfect representation of the Middle East region with all their heterogenous backgrounds.

## Subjects and methods

This study was conducted over a 1-year period from February 2012 to February 2013 in Hamad General Hospital outpatient clinics and primary healthcare centres. The study was approved by the Institutional Review Board (11,250/2011) of the Institute’s Medical Research Center.

### Subjects

A total of 4000 males were invited to be included in the study. Subjects encompassed male patients, their companions, in addition to regular visitors to our hospital. All these gentlemen were entitled to answer questionnaires supplied in both the Arabic and English languages. Each of the participants met a co-investigator in a private setting to describe the nature and benefits of the study and to explain how to complete the questionnaire. All subjects were assured that the collected information would remain confidential and would not include any personal data except age, nationality, and marital status. The option to participate or to refrain from the study was given to each participant.

The inclusion criteria were adult married males from various age groups without age restrictions. The study excluded: (I) single males, (II) patients with paraplegia or neurological diseases that may adversely affect ejaculation (e.g. severe peripheral neuropathy and multiple sclerosis), (III) patients receiving medications that may affect ejaculation, such as antidepressants and antipsychotics.

### Questionnaires

Each patient was asked to complete two separate questionnaires. The first was a self-report questionnaire that was formulated by the research group. It started with identifying participant age, nationality, and marital status. It included six questions meant to assess self-reported perception of PE. The questions enquired about whether the subject presumes he has PE or not, duration of the condition if present, and whether he had normal ejaculation before starting to develop PE or not. In addition, subjects were asked about the time it usually takes between intromission and ejaculation (actual IELT [IELT-a]) and the time they assumed to be the normal average (perceived IELT [IELT-p]). Then it asks if the patient ever consulted his physician for the complaint of PE or had received any medications for the treatment of PE. Eventually, this part asks if the participant has erectile dysfunction or is suffering from any chronic medical illness (Appendix A).

The second questionnaire was the AIPE, which has been proven to be a valid patient-related outcome measure in diagnosing PE [4]. The AIPE is composed of seven questions each of them is scored on a 5-point ordinal scale in which a lower score means poorer sexual function. Subjects were asked to rate their sexual desire, sexual potency, IELT time, their ability to prolong IELT, sexual satisfaction for the person in addition to his partner and finally how often sexual intercourse is associated with much stress or anxiety (Appendix B).

### Data collection

Patients reporting PE in the self-report questionnaire were labelled the PE group. Patients who did not complain of PE were labelled as the No-PE group. The reported IELT-a, as well as the IELT-p was recorded. Patients in the PE group were subclassified according to age into four sub-groups (<30, 30–40, >40–50, >50 years). Results of the AIPE questionnaire were recorded in the PE group and patients were re-classified accordingly into AIPE-PE (AIPE Score ≤30) and AIPE-No-PE (AIPE Score >30) groups.

### Statistical analysis

The data from each questionnaire was extracted to a data sheet for statistical analysis. Data was evaluated qualitatively and quantitatively. All statistical analyses were done using the Statistical Package for the Social Sciences (SPSS®), version 18.0 (SPSS Inc., Chicago, IL, USA). Descriptive statistics were used for demographic and clinical patients’ characteristics. Numbers (frequencies) were used to report categorical variables, while median (interquartile range [IQR]) was used to display numerical variables. Prevalence of outcome was reported using *Z*-test. The chi-square test was used to examine qualitative measurements for associations between different factors and the outcome. Spearman correlation analysis was used for assessing the relationship between quantitative variables. The Kruskal–Wallis test was used to compare the reported and presumed IELT between different AIPE scores. The Mann–Whitney test was used to compare different numerical variables between participants in the AIPE-PE and AIPE-No PE groups.

## Results

We offered our questionnaire to 4000 candidates and 560 did not want to participate. Of the 3440 responders, 3042 candidates fit the inclusion criteria. The mean (SD) age of the participants was 37.09 (9.1) years. In all, 321 were Qatari nationals (10.6%) and 2721 were expats from different nationalities (89.4%). The median (IQR) IELT-a in the whole sample was 5 (3–13.5) min, while the median (IQR) ILET-p was 15 (5–15) min. The median (IQR) AIPE score for the whole sample was 27 (23–29). A total of 2550 of the participants had no known medical comorbidities (83.8%), while 492 had known chronic medical conditions (16.2%) including 267 with diabetes mellitus (DM; 8.8%) and 119 were hypertensive patients on treatment (3.9%). A total of 51 participants had both DM and hypertension (1.7%).

The prevalence of PE by self-report was 38.5% (1171/3042 men), of which 32% reported to have PE from their initial sexual activity (lifelong PE) (375 men), while 68% had experienced PE after a period of normal ejaculation (acquired PE) (796 men). Table 1 shows the difference between the PE and No-PE groups. There were significant differences between the groups as regards IELT-a, AIPE score and IELT-p (all *P* < 0.001).

**Table 1.** Difference between the PE and No-PE groups by self-report

	PE Group (*n*= 1171)	No-PE Group (*n*= 1871)	*P*
Age, years, median (IQR)	34 (30–42)	36 (30–42)	0.2
IELT-a, min, median (IQR)	3 (2–10)	6 (4–15)	<0.001
AIPE score, median (IQR)	25 (21–27.5)	28 (25–30)	<0.001
IELT-p, min, median (IQR)	15 (10–20)	15 (5–15)	<0.001

Among the PE group, the median (IQR) age was 34 (30–42) years, 14.6% of them (171 men) were Qatari nationals and 85.4% were from other nationalities (1000 men). Only 36% of this group (422 men) sought medical advice for PE and 27.2% (319 men) received treatments for the condition; 77.4% of the medications were prescribed by physicians (247 men) and 22.6% were over the counter medications (72 men). In all, 204/319 patients who received PE medications complained of acquired PE.

In the PE group, the median (IQR) AIPE score was 25 (21–27.5). Table 2 shows the severity variations in the AIPE score for the PE Group; 1102 participants (94.1%) had AIPE-PE, while 69 (5.9%) had AIPE-No PE. Most cases were mild or mild to moderate (76.6%).

**Table 2.** Results of the AIPE score among self-reported PE group

	AIPE score	Prevalence, *n* (%)
Severe PE	7–13	27 (2.3)
Moderate PE	14–19	178 (15.2)
Mild to moderate PE	20–25	526 (44.9)
Mild PE	26–30	371 (31.7)
No PE	31–35	69 (5.9)

Within the PE Group, 609/1171 participants reported their IELT-a, and the median (IQR) was 3 (2–10) min. In all, 559/1171 participants described the IELT that they presumed to be normal, and the median (IQR) IELT-p was 15 (10–20) min. According to the AIPE severity classification, the differences in IELT-a and IELT-p between the severity groups were highly significant, with the duration of both IELT-a and ILET-p being higher in the AIPE-No-PE and Mild-PE groups (*P* < 0.001 for both; Table 3).

**Table 3.** Actual and presumed IELT among different severity groups

PE sub-grouping according to severity	IELT-a	IELT-p	P*
Severe PE, median (IQR)	2 (1–2)	15 (10–20)	<0.001
Moderate PE, median (IQR)	2 (1–3)	15 (10–15)	<0.001
Mild to moderate PE, median (IQR)	3 (1–5)	15 (10–15)	<0.001
Mild PE, median (IQR))	10 (3–20)	15 (10–30)	<0.001
AIPE-No PE, median (IQR)	13 (8–20)	20 (15–52.5)	<0.001

*Kruskal–Wallis test.

**Figure 1.**
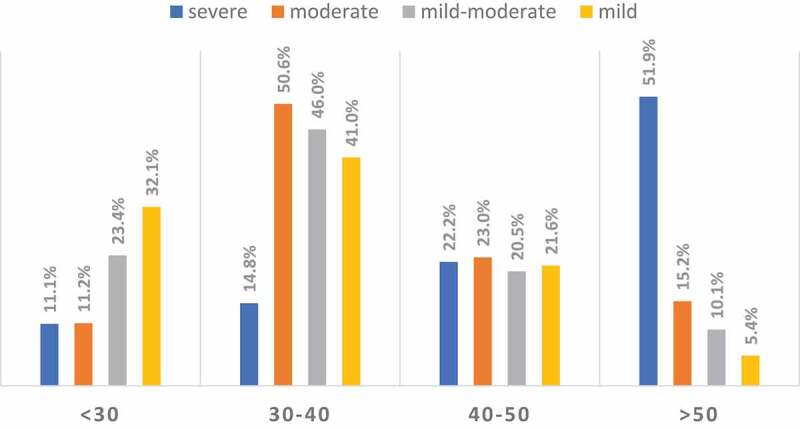
Distribution of the AIPE scores according to age in men in the PE Group

For the sub-classification according to age (<30, 30–40, >40–50, >50 years), a negative correlation was found between the AIPE score and age in the PE group (*r–* 0.254; *P*< 0.001) with increasing prevalence of severe PE with older age groups (Figure 1).

Using the AIPE questionnaire, 1102/1171 participants were found to have AIPE-PE, while the remaining 69 were not found to have PE (AIPE-No PE). Table 4 shows the difference between men with diagnosed PE and No-PE according to the AIPE score within the PE group. The participants’ age was significantly higher in the AIPE-PE group. The IELT-a and IELT-p were significantly higher in the AIPE-No-PE group. There were significant differences between the two groups for the individual questions of the AIPE 3, 4 and 5–7, while no significant difference could be found between both groups as regards questions 1 and 2.

**Table 4.** Within the PE group: difference between diagnosed PE and No-PE according to AIPE score

	AIPE-PE, *n*= 1102	AIPE-No-PE, *n*= 69	*P*
Age, years, median (IQR])	35 (31–42)	33 (22.5–35)	<0.001
IELT-a, min, median (IQR) [*n*= 609]	3 (2–10) [*n*= 559]	13 (8–20) [*n*= 50]	<0.001
IELT-p, min median (IQR) [*n*= 559]	15 (10–20) [*n*= 509]	20 (10–48.75) [*n*= 50]	<0.001
Lifelong, *n* (%)	356 (32.3)	19 (28)	0.247
Acquired, *n* (%)	746 (67.7)	50 (72)	
AIPE score, median (IQR)	24 (21–27)	31 (31–32)	<0.001
Q1	4 (3–4)	5 (4–5)	0.21
Q2	4 (3–4)	5 (4–5)	0.48
Q3	4 (2–5)	4 (4–5)	<0.001
Q4	3 (2–4)	5 (4–5)	<0.001
Q5	3 (2–4)	5 (4–5)	<0.001
Q6	3 (2–4)	5 (4–5)	<0.001
Q7	4 (3–4)	5 (4–5)	<0.001

Q, Question.

## Discussion

The present study was conducted on 3092 participants aiming at evaluating the prevalence of PE among the married gentlemen in Qatar using both, self-reporting and the validated AIPE questionnaires. In addition, this study targeted evaluation of the population perception of normal IELT and its reflection on the complaint of PE.

The prevalence of the complaint of PE by self-report in Qatar was found to be 38.5%. This result was higher than the self-reported prevalence in Argentina 28.3% [8], Turkey 20% [9], and South Korea 19.5% [10], using direct interview. On the other hand, this result was much less than the self-reported results in the USA (77.6% [11]) and Middle East (82.6% [12]) using online surveys.

In the present study, when assessed objectively using the AIPE score, the prevalence of PE was found to be 36.2%, with most cases being mild or mild-to-moderate severity encompassing 44.9% of the cases. This prevalence was higher than that reported by other studies using a PEDT score of ≥11 as an objective measure to diagnose PE, where the prevalence of PE was reported to be 26.67% in Egypt [13], 16% in the Asia Pacific region [14], and 11.3% in South Korea [10]. However, it was less than the prevalence reported in the USA (with PEDT of ≥11), which was 49.6% [11], and less than the prevalence reported in the Kingdom of Saudi Arabia (KSA) of 50.8% using the DSM-IV definition of PE [15].

More recent studies applied the Waldinger and Schweitzer definition of PE and the four subtypes they proposed which are lifelong, acquired, subjective, and variable PE [1]. Serefoglu et al. [9] investigated the prevalence of PE in 17 provinces in Turkey in a non-interventional, observational, cross-sectional field survey. That study reported the prevalence of the four subtypes of PE to be: lifelong PE, 2.3%; acquired PE, 3.9%; variable PE, 8.5%; and subjective PE, 5.1%; with total prevalence of 19.8%. Gao et al. [16] studied 3016 Chinese male patients complaining of PE. The study tool was face-to-face interviews during which subjects were required to answer a verbal questionnaire. The study found the prevalence of lifelong PE to be 3.18%, acquired PE 4.84%, variable PE 11.38%, and subjective PE 6.4%, with a total prevalence of 25.8%. In the present study, we subdivided patients with PE as lifelong PE comprising patients who complained of PE throughout their sexual intercourses from their first sexual activity and acquired PE, describing those patients who developed PE after initially having normal ejaculation. We noticed a greater prevalence of acquired PE (68% of the PE complaints), which agrees with previous studies.

The IELT has been assessed by different researchers with conflicting results. In a large study by Waldinger et al. [17], the IELT was assessed across five countries with an overall median (IQR) value of 5.4 (0.55–44.1) min. A variation between countries was found with the median (IQR) value for Turkey being 3.7 (0.9–30.4), Spain 5.8 (2.3–15.3), Netherlands 5.1 (0.5–33), UK 7.6 (1.7–42.3), and USA 7 (0.7–44.1) min, respectively. These data are comparable to the present study where the median (IQR) IELT-a of the total study population was 5 (3–13.5) min by self-report.

The difference seen between our present results and the previous reports can be attributed to multiple factors. PE prevalence has shown variability amongst different ethnic and cultural backgrounds [6,10,13,14]. The conservative culture in Middle East countries and lack of sexual education may play a role in increasing the prevalence of PE in the area. More importantly, the definition of PE adopted by each study is expected to have a major effect on defining patients with PE. For Example, Shaeer et al. [11] assessed the prevalence of PE in the USA using self-report, PEDT and ISSM definition of PE with results of 77.6%, 49.6% and 6.3%, respectively. There was a significant difference between the PEDT and ISSM prevalence. Also, the method used for collecting data can have its impact on the result reported. Face-to-face interview is considered more accurate than an online survey. This is extremely valuable for full evaluation of men with PE as it will provide the needed help and explanation to clear any doubts for the study population [3].

The present study is the first to assess the population perception of normal IELT. Interestingly, overall study subjects had a median (IQR) estimation of IELT-p of 15 (10–20) min. A large difference can be found between the median (IQR) IELT-a vs IELT-p, at 5 (3–13.5) vs 15 (5–15) min. The wide gap between the IELT-p and the IELT-a can be attributed to the cultural background. Middle Eastern countries tend to have lack of knowledge as regard sexual relations, which is mainly attributed to a lack of proper sex education in schools and the conservative culture of the community. The IELT-p was found to be higher in the self-reported PE group than the non-PE group, with a median (IQR) of 15 (10–20) vs 15 (5–15) min. This reflects a more realistic expectations from men not suffering from PE than those who self-report having PE. The exaggerated expectation by the PE group may have led to anxiety and dissatisfaction, which will eventually lead to a PE complaint. This exaggerated expectation may mainly be due to lack of sex education. Whether this difference plays a role in the increased prevalence of self-reported PE or not, is a subject that needs further evaluation. Another interesting finding was the significantly increased IELT-p in candidates with mild or no PE according to the AIPE score. These men although having no or a mild PE problem, their unrealistic expectation of a long IELT-p stressed them into believing that they had PE.

Interestingly, in the present study, 5.9% of men who self-reported that they had PE were diagnosed as no PE with the AIPE score. Their AIPE score was significantly higher than those with AIPE-PE as regards the total score as well as in the individual questions of ejaculation domain (questions 3 and 4) and satisfaction domain (questions 5–7), while no significant difference could be found between both groups as regards erection domain (questions 1 and 2). Although the IELT-a was significantly higher in the AIPE-No-PE group than the AIPE-PE group, the IELT-p was also significantly higher in the AIPE-No-PE group. This explains why these candidates are convinced that they have PE although their scores are within the normal range. Their expectations are high and unrealistic about normal ejaculation and thus they are stressed and may seek treatment for PE.

The lack of proper sex education, associated with the psychological impact of diminished self-confidence and sexual skills, can have a drastic impact on the quality of sexual life. The vicious circle of interpersonal stress affecting IELT resulting in more performance anxiety can be associated with more symptoms of PE and other sexual complaints. Recently, education and reassurance have been recommended for men with variable PE. Patients with subjective PE may require referral to psychotherapy [18]. Unfortunately, most of the studies that assessed educational interventions and psychotherapeutic treatment for PE were not able to reach strong conclusions. Most of these studies were unblinded and lacked control. More research is needed to assess the role of sex education and behavioural modification in the management of PE.

In the present study, it was found that the AIPE score was negatively correlated with age indicating a greater severity of PE in the older population. This was also reported by other studies on Turkish men [9] and Italian men [19]. Waldinger et al. [17] also reported that IELT decreased with age, with the shortest IELT seen in men aged ≥51 years. On the other hand, Hanafy et al. [13] in 2019 reported an insignificant difference for age on PE on his study done on Egyptian men using the PEDT.

Men with acquired PE are more likely to seek medical treatment compared to patients with life-long PE. This may be explained by the fact that them being previously normal alerts them more and causes more stress to seek treatment [9,16]. This observation was also found in the present study were 204/319 patients who received PE medications complained of acquired PE.

The present study used direct interview of patients with explanation of any doubts as regard the questions included in both the questionnaires. This is considered to add to the accuracy of the data collected than using online questionnaires. Also, the present study is the first in the Middle East to use a large study population for evaluating the complaint of PE and its associations.

There are some limitations to the present study. First, Qatar is a cosmopolitan country with most of the population expats from various cultural backgrounds. As previously mentioned, the prevalence of PE is variable among various cultural backgrounds. Thus, although the study sample is representative of the population of Qatar, the responses are not related to a single homogenous cultural background. Also, the IELT reported in the study was self-estimated and not directly measured using a timer. In addition, having subjects in the study population away from their families and not having regular sexual activity may have an impact on the responses received. Moreover, the present study was conducted through 2012 before the release of the more recent definition of PE by the ISSM and DSM-V in 2013. The objective part of the present study was based on the AIPE score, which was formulated depending on the criteria set by the second consultation on sexual dysfunctions that considered an IELT of ≤2 min to be one of the criteria for a diagnosis of PE [4]. More recently, the DSM-V and the ISSM have adopted new definitions for lifelong and acquired PE. These definitions correlate lifelong PE with an IELT of ≤1 min and acquired PE with an IELT of ≤3 min. This update in definition should have its impression on the results reported [1,20].

## Conclusion

The present observational, non-interventional, epidemiological cross-sectional study found a prevalence of PE in the male population of Qatar of 38.5% by self-report, while it was 36.2% using the AIPE score. PE prevalence was found to increase with age. The present study is the first to assess the perceived normal IELT in patients with a complaint of PE and it was found to correlate with the complaint of PE. Overall, PE is one of the most common sexual dysfunctions in the Middle East as worldwide. More studies are required to thoroughly investigate the magnitude of PE and its impairment on sexual life in the Middle East.

## References

[1] Serefoglu EC, McMahon CG, Waldinger MD, et al. An evidence-based unified definition of lifelong and acquired premature ejaculation: report of the second International Society for Sexual Medicine Ad Hoc Committee for the Definition Of Premature Ejaculation. Sex Med. 2014;2:41–59. 25356301 10.1002/sm2.27 PMC4184676

[2] Saitz TR, Serefoglu EC. Advances in understanding and treating premature ejaculation. Nat Rev Urol. 2015;12(11):629–640. 26502991 10.1038/nrurol.2015.252

[3] Althof SE. Patient reported outcomes in the assessment of premature ejaculation. Transl Androl Urol. 2016;5(4):470–474. 27652219 10.21037/tau.2016.05.04 PMC5001997

[4] Arafa M, Shamloul R. Development and evaluation of the Arabic Index of Premature Ejaculation (AIPE). The Journal of Sexual Medicine. 2007;4(6):1750–1756. 17970977 10.1111/j.1743-6109.2006.00213.x

[5] Saitz TR, Serefoglu EC. The epidemiology of premature ejaculation. Transl Androl Urol. 2016;5(4):409–415. 27652213 10.21037/tau.2016.05.11 PMC5001986

[6] Nicolosi A, Laumann EO, Glasser DB, et al. Sexual behavior and sexual dysfunctions after age 40: the global study of sexual attitudes and behaviors. Urology. 2004;64(5):991–997. 15533492 10.1016/j.urology.2004.06.055

[7] Lauman EO, Nicolosi A, Glasser DB, et al. Sexual problems among women and men aged 40–80 y: prevalence and correlates identified in the Global Study of Sexual Attitudes and Behaviors. Int J Impot Res. 2005;17(1):39–57. 15215881 10.1038/sj.ijir.3901250

[8] Nolazco C, Bellora O, López M, et al. Prevalence of sexual dysfunctions in Argentina. Int J Impot Res. 2004;16(1):69–72. 14963474 10.1038/sj.ijir.3901140

[9] Serefoglu EC, Yaman O, Cayan S, et al. Prevalence of the complaint of ejaculating prematurely and the four premature ejaculation syndromes: results from the Turkish Society of Andrology Sexual Health Survey. J Sex Med. 2011;8(2):540–548. 21054799 10.1111/j.1743-6109.2010.02095.x

[10] Lee SW, Lee JH, Sung HH, et al. The prevalence of premature ejaculation and its clinical characteristics in Korean men according to different definitions. Int J Impot Res. 2013;25(1):12–17. 22931761 10.1038/ijir.2012.27

[11] Shaeer O. The global online sexuality survey (GOSS): the United States of America in 2011 Chapter III--Premature ejaculation among English-speaking male Internet users. J Sex Med. 2013;10(7):1882–1888. 23668379 10.1111/jsm.12187

[12] Shaeer O, Shaeer K. The Global Online Sexuality Survey (GOSS): male homosexuality among Arabic-speaking internet users in the Middle East--2010. J Sex Med. 2014;11(10):2414–2420. 25060943 10.1111/jsm.12634

[13] Hanafy S, Hamed AM, Hilmy Samy MS. Prevalence of premature ejaculation and its impact on the quality of life: results from a sample of Egyptian patients. Andrologia. 2019;51(8):e13298. 31025424 10.1111/and.13298

[14] McMahon CG, Lee G, Park JK, et al. Premature ejaculation and erectile dysfunction prevalence and attitudes in the Asia-Pacific region. J Sex Med. 2012;9(2):454–465. 22023395 10.1111/j.1743-6109.2011.02507.x

[15] El-Sakka AI. Association of risk factors and medical comorbidities with male sexual dysfunctions. J Sex Med. 2007;4(6):1691–1700. 17081221 10.1111/j.1743-6109.2006.00342.x

[16] Gao J, Zhang X, Su P, et al. Prevalence and factors associated with the complaint of premature ejaculation and the four premature ejaculation syndromes: a large observational study in China. J Sex Med. 2013;10(7):1874–1881. 23651451 10.1111/jsm.12180

[17] Waldinger MD, Quinn P, Dilleen M, et al. A multinational population survey of intravaginal ejaculation latency time. J Sex Med. 2005;2(4):492–497. 16422843 10.1111/j.1743-6109.2005.00070.x

[18] Althof SE, McMahon CG, Waldinger MD, et al. An update of the international society of sexual medicine’s guidelines for the diagnosis and treatment of premature ejaculation (PE). Sex Med. 2014;2(2):60–90. 25356302 10.1002/sm2.28 PMC4184677

[19] Verze P, Arcaniolo D, Palmieri A, et al. Premature ejaculation among Italian men: prevalence and clinical correlates from an observational, non-interventional, cross-sectional, epidemiological study (IPER). Sex Med. 2018;6(3):193–202. 29803639 10.1016/j.esxm.2018.04.005 PMC6085227

[20] American Psychiatric Association. Diagnostic and Statistical Manual of Mental Disorders, 5th edition (DSM-5). Washington, DC: American Psychiatric Association; 2013.

